# Academic collaboration with industry: Case studies from health care delivery operations

**DOI:** 10.1007/s10729-026-09798-y

**Published:** 2026-11-11

**Authors:** Craig M. Froehle, Wiljeana Glover, Xin David Ding, Vikram Tiwari

**Affiliations:** 1 University of Cincinnati, 2906 Woodside Dr, Cincinnati, OH 45221-0130 USA; 2 Babson College, Wellesley, MA USA; 3 Rutgers University, Newark, NJ USA; 4 Vanderbilt University, Nashville, TN USA

**Keywords:** Academia-industry collaboration, Health care, Healthcare, Education, Case study

## Abstract

Business and engineering scholarship's relevance largely relies on its practical applicability. As health care delivery grows to represent a major portion of the global economy, its distinct culture and unique challenges necessitate stronger relationships between health care practice and academia. This paper explores academic collaborations with the health care delivery industry, emphasizing the compelling benefits for health care organizations (and their patients), researchers, and students in the context of care delivery operations. Through four case studies, and by drawing upon our experiences as a diverse set of operations scholars, we highlight successful models of collaboration between operations academia and health care institutions. These models enable many of the benefits realized, such as reductions in patient wait times and visit lengths, more equitable physician scheduling, faster ambulance dispatch, and reduced surgical appointment cancellations, among others, while also improving relevance for students and practical application for theory. While the potential benefits are clear, challenges persist, often emanating from cultural differences, funding uncertainties, and the nuances of industrial vs. academic objectives. However, when navigated effectively, these collaborations can advance both sectors, bridging the gap between academic theory and real-world application. Drawing from existing literature and supplemented by rich case study evidence, this article offers guidance for care institutions and individual scholars seeking to establish and foster similar partnerships. By delineating the parameters of successful collaborations and drawing from a diverse portfolio of experiences, this paper hopes to encourage and enable more synergistic efforts, potentially transforming health care operations and leading to improved outcomes for patients, institutions, and academia.

## Highlights

• The article presents four unique case studies and multiple other examples from the literature to explore successful collaborations between academic institutions and health care delivery organizations. These case studies and examples provide insights into the collaboration mechanisms, challenges faced, and outcomes achieved.

• The collaborations detailed in the article have led directly to improvements in health care delivery operations, such as more efficient patient scheduling, better resource allocation, and enhanced care delivery performance. This framework is valuable for health care managers, clinicians, and care organizations looking to improve their operations, systems, and patient outcomes.

• The paper identifies several characteristics of successful collaboration, such as clear communication and alignment of goals between academic and industry partners. We emphasize the importance of stakeholder engagement, which is crucial for managers, academics, and policy-makers aspiring to foster effective partnerships.

• Several of the collaborative models described here offer significant educational opportunities for students, providing them with practical experience in real-world health care operational settings. This benefits academic researchers and educators by preparing students for careers, both applied and scholarly, in healthcare operations and management.

• By engaging in these collaborations, both academia and industry benefit from bridging the gap between scholarly theory and practical application. This is particularly valuable for healthcare delivery organizations aiming to implement evidence-based practices and innovations in their operations.

## Introduction

Operations scholarship, encompassing both research and education, is fundamentally concerned with its relevance to industry and utility to practice. This sentiment has been stated many times over the years (e.g., [1–3]). In 1980, Buffa clearly expressed this belief when saying, “…we should take our objectives and criteria set from real systems” ([4], p. 1). Some forty years later, Kc et al. (2020) again emphasized “a call to action for greater engagement with the medical community in areas where tools and insights of operations management can bring about improvements in healthcare delivery” ([5], p. 73). Also, recently, Carter & Busby (2023) asked the following: “Given the state of healthcare systems across the globe, there should be a larger role for OR in transforming care. So why has this not occurred? Why is it such a challenge to have a large-scale impact?” ([6], p. 1060). The reiteration of this call for a larger and more productive relationship with the healthcare delivery industry after such a time gap suggests it is not readily achieved, despite the many benefits of doing so.

Reflecting upon over five collective decades of experience working directly and closely with healthcare delivery institutions, the authors can conclude only that collaboration with industry partners can be an immensely effective and fulfilling way to improve this scholarship. Such partnerships benefit organizations by providing access to specialized resources; benefit their patients by improving the quality, consistency, and accessibility of care; benefit researchers by offering problem-oriented insights, specialized data, and contemporary environments; and benefit students by better preparing them for relevant professional careers. In fact, many healthcare-related operations and management science papers simply would not be possible without significant collaboration with a healthcare organization (e.g., [7–16]).

However, a persistent barrier to collaboration we often hear expressed by operations academics themselves is the lack of ongoing relationships with industry partners, which can be difficult to identify, establish, and scale. Our experience suggests that ongoing relationships can reduce search and startup costs for faculty, improve communication and outcomes, and expand opportunities because, if for no other reason, each party better understands the needs and capabilities of the others.

We have found that the relationship connecting business schools to health care tends to be substantially different from other academic partnerships, such as those in the biological sciences, medicine, or public health, which are often more economically co-dependent through mechanisms such as grants, patents, and commercialization [17, 18]. Devising collaborative programs where all parties benefit takes significant, ongoing effort and requires a shared understanding of each participant's goals and capabilities. This can be particularly challenging in health care, where partnerships may be hindered by factors such as discrepancies between objectives and resources, conflicting reimbursement systems, and limited planning [19].

The healthcare industry, representing a significant portion of the global economy (e.g., roughly one-fifth of the US GDP and over 10% of the EU27 GDP [20]), can require a deep well of specialized knowledge and is fairly insular, with most professionals arriving into it via dedicated educational channels. Despite calls for integrating operations knowledge and experience outside of medicine into clinical systems and operations (e.g., [21, 22]), there are few long-running, systematic efforts across academia, the healthcare delivery industry, or any major world government to establish such a sweeping initiative (although the prevalence of general business training in health care has improved over the last couple of decades, at least in some countries) [23]. One notable effort to narrow this gap was the US Department of Veteran Affairs (VA) establishing four regional Engineering Research Centers (ERCs) focused on process improvement. The New England Veterans Engineering Resource Center (NE-VERC), successfully engaged academic industrial engineering departments to create research and fellowship opportunities (e.g., [16]).

While success stories can be found, we feel the healthcare industry stands to benefit greatly from enhanced and expanded collaboration with academia, especially in operations management, and vice versa [24]. Healthcare organizations face significant challenges related to efficiency, quality, and employee well-being that can be addressed by operations analysis [25, 26]; yet, we have found that business and engineering students tend to have limited exposure to the healthcare industry beyond any experiences they may have had as patients. This lack of exposure offers them a limited view into that industry’s systems, practices, and culture, and can lead to assumptions that health care’s operational problems are similar, if not identical, to their own or to other service industries (assumptions that are often inaccurate and, sometimes, fundamentally wrong). And, given that many of the operational innovations in the health care industry have come from the industry itself, academics and students have the additional benefit of being able to collaborate with and learn from intelligent, motivated professionals eager to improve the care they provide and the organizations and processes in which they operate.

Given the potential that improving healthcare operations has for positively affecting people's lives, such collaborations can be highly fulfilling. We feel both health care and business/engineering academia would benefit from having access to pre-existing collaboration models that have been successfully designed and executed. This paper uniquely aims to provide such insights, spanning research and education to benefit scholars and students across the spectrum of operations academia, thereby facilitating the further integration of operations research and management in health care through long-term, impactful collaboration.

In the next section, we briefly highlight and synthesize some of the limited literature on academic-industry collaborations (sometimes referred to in the literature as AICs or as university-industry collaborations), with a specific emphasis on the structure and management of them within the health care delivery. Notably, we do not attempt to catalog and analyze the myriad papers that merely *mention* collaboration between care delivery organizations and academia, or the various papers resulting from such joint efforts, implementations, and/or practice-motivated problems, as it would be unwise to attempt such an undertaking given the many thousands of examples that likely exist [20]. Rather, we briefly examine a selected set of papers that focus *specifically* on understanding how such collaborations *in general* can be more effective. Following that, we explore four detailed case studies of successful collaboration models, as well as overviews of several additional documented approaches, including selected examples of problem-focused papers that discuss a collaborative effort in substantial detail, supplemented with our own insights. In the final section of the paper, we use the inductive reasoning approach to synthesize additional insights from the case studies, then offer general conclusions and prescriptive guidance for those aspiring to establish and/or sustain such collaborative programs of research, study, and operational improvement. We end the paper with a general call for operations researchers to invest in these collaborative efforts in order to enhance both the usefulness and theoretical relevance of our field’s scholarly endeavors.

## Brief synthesis of the academic-industry collaboration literature

Broadly taken, academic-industry collaborations refer to the interactive efforts between higher educational institutions and industry that principally encourage knowledge sharing, cooperative problem-solving and improvement, and technology exchange [27]. When thinking specifically about these collaborations in the context of health care delivery, the literature that focuses on how and why they succeed (or fail) is fairly limited. In this section, we borrow some ideas from the broader literature, but focus primarily on the lessons learned from health care delivery-related collaborations. We find that while the lessons learned are myriad, they align along four core themes, which are consistent with our experience as well.

### Prioritizing relevance and real-world impact

This first theme is typically offered as the motivation for literature reviews and agenda-setting articles on this topic, just as it is in this paper. It is often stated that relevance to practice is a *raison d'être* of the broader operations field [3] and health care operations in particular [1, 25]. While we will not reiterate here the case made in these papers, for we ourselves make it in the introduction to this article, suffice to say that relevance, real-world impact, and driving improvement in industry are widely agreed as valuable, if not primary, goals for our field. Such real-world impact may be illustrated by implementing novel processes or digital innovations, disseminating shared know-how, or new intellectual property from the collaboration [28]. And, despite the difficulty in executing such collaborations [27], they can be transformative.

### The necessity of strong relationships

In order to effectively collaborate with the health care delivery industry, we need to foster and maintain strong relationships that lead to and amplify meaningful interdisciplinary exchange [26, 29]. Effective research, particularly in complex settings like health care delivery, is inherently collaborative and interdisciplinary, requiring operations scholars to forge relationships with practitioners and industry leaders [1, 14]. Domain experts on both sides are crucial for identifying and framing important problems (versus assuming a solution and finding someone to merely design and implement it, which is an all-too-common first conversation with some health care practitioners who assume they know the root cause of their operational issue), understanding salient aspects, acquiring necessary data, validating models, and disseminating results. Such high-functioning partnerships require strong relationships, solid interpersonal dynamics, and, above all, mutual trust [29]. This is similar to the idea of a “strong champion” as advocated by [6], but broader in scope. Successful projects require a genuine commitment from both sides, often necessitating significant time investment and physical presence by academics to acquire expert knowledge about the processes and procedures in place [3]. In our experience, obtaining such a commitment can be difficult (if not impossible) without strong interpersonal relationships being in place first.

### Managing expectations and communication

A recurring sentiment in this literature, especially emphasized in health care contexts, is that misalignment in goals, expectations, and communication styles can pose a substantial barrier to collaboration success [28–30]. This misalignment can occur in part from the need to balance both operations research-related knowledge with health care knowledge [16]. From the outset of any collaboration, shared clarity regarding roles, goals, and outcomes is a must ([14]. Key obstacles include ambiguous goals, incompatible timelines (in our experience, industry tends to want to move more quickly than academia is able to support), funding and resource contributions, and conflicting priorities (e.g., scientific vs. business). Transparency and shared, unambiguous terminology are essential [29], regardless of who initiates the collaborative effort, and failure to recognize factors that are important to decision-makers, even those that may be difficult to operationalize, can hinder adoption of any improvement plans or solutions that are generated [1]. This is especially true when the academic side brings in novel theories, tools, or methods that the industry side may not inherently understand or trust [31].

### Satisfying Conflicting Cultures and Support Systems

Academics and industry partners tend to operate under different institutional norms and reward structures. The deliverables and outcomes of academic-industry collaborations with health care delivery organizations can include (some examples as cited) research articles and white papers, technology and patents [32], institutional and care process changes, staff training and education programs, and a variety of other work products. But scholars working on impactful, real-world problems face a dual burden: they must support their healthcare partners working in complex, operationally constrained systems that practically seek improvement not optimization, they must publish in medical/health journals read by decision-makers to influence practice, *and* simultaneously they must publish in theory-valuing operations journals to disseminate technical results and meet their academic institutions’ expectations [1, 14, 26]. This often results in collaborative teams splitting in two, with health care team members writing for their audience and the operations scholars writing for theirs [33]. These collaborations also face the risk of overspecializing, which can harm both the generalizability of the findings and the ability of the academic team members to work effectively on a portfolio of research [34]. In this literature, the result of these concerns is often a call for action; specifically, papers will lament and/or challenge the burden and barriers that tenure and publishing expectations create for collaboration-minded academics [1]. But journals and funding agencies also receive a fair amount of the blame, with accusations of being overly fixated on theoretical rigor when deeply practical may actually have a bigger impact on health care delivery and societal well-being [1]. As a result, some call for senior faculty, who have no further promotions to seek, to take the lead in establishing such collaborative relationships [3]. More broadly, there are initiatives to reshape promotion, tenure, publication, and funding expectations to more closely align with impact and less with the narrower, theory-focused value set historically used in operations academia (e.g., [35]; and the San Francisco Declaration on Research Assessment, or DORA, which spans academic fields).

In the next section, we provide detailed accounts from four successful collaborations between universities and health care entities. Guided by the themes emphasized in this literature synthesis, we discuss each of the four case studies along four topics: Primary Goals, or the motivation and/or genesis of the collaboration; Collaborative Structure, or the organizational and logistical nature of the relationship; Project Effectiveness and Outcomes, or the results of the collaboration in terms of deliverables and meeting its original goals; and Lessons Learned, or the wisdom and insights about these endeavors we have gained along the way. The case studies are necessarily summarized, so some details will have been omitted. While these cases range from traditional “cross-campus” arrangements to complex international undertakings, they all lasted considerable periods of time and produced substantially positive outcomes for most, if not all, of the entities involved.

## Case studies

**Fig. 1:**
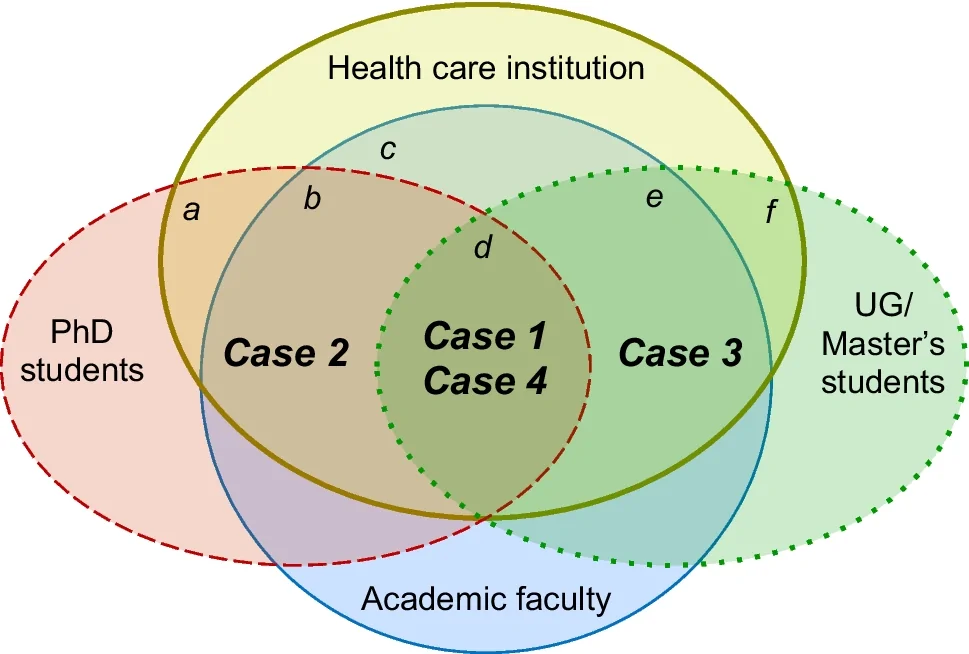
Involved parties for collaborative case studies and models discussed here. Case 1: Cancer Center Institute of New Jersey (Rutgers-CINJ); Case 2: University of Cincinnati Emergency Medicine (Cincinnati); Case 3: Global Health Innovation Lab-Babson College (GHIL-Babson); Case 4: Michael E. DeBakey VA Medical Center in Houston, Texas (MEDVAMC); Zones a-f are described further in §3.5

In the evolving landscape of healthcare operations, academic collaborations with industry are becoming increasingly vital to drive innovation, improve efficiency, and enhance patient care. In this section, we present (in a necessarily abridged fashion) four case studies illustrating successful partnerships between academic institutions and healthcare organizations. While each case study considers a wholly unique approach and context, each also provides unique insights into the collaboration mechanisms, challenges faced, and outcomes achieved. Together, these case studies underscore the transformative potential of academic-industry collaborations in addressing complex healthcare challenges and fostering educational growth. To help organize these case studies, as well as other collaborative models discussed here, in a comprehensive framework, we offer Fig. 1, which identifies the parties of primary importance to the purpose and function of each approach.

### Case study 1 – Cancer Institute of New Jersey (Rutgers-CINJ)

The Cancer Institute of New Jersey (CINJ), designated as one of only 51 Comprehensive Cancer Centers (CCC) by the National Cancer Institute, aimed to address patient scheduling issues and long waiting times in their oncology outpatient units before moving to a new facility in 2025. CINJ is part of UMDNJ-Robert Wood Johnson Medical School (RWJ) and the only CCC in New Jersey, USA. CINJ provides adult and pediatric patients with access to the most advanced cancer treatment options including clinical trials, precision medicine, immunotherapy, complex surgical procedures, and sophisticated radiation therapy techniques. It manages more than 100,000 patient visits per year. Key stakeholders in this project included leading physicians, nursing officers, directors, and nurse practitioners from RWJ, as well as faculty members, Ph.D. students, and master’s students from the MS in Healthcare Analytics and Intelligence program at Rutgers Business School.

#### Primary goals

Primary goals included (a) providing supply chain Ph.D. students with opportunities to apply simulation and analytical modeling techniques to real-world clinical scheduling bottlenecks, (b) enabling master’s students to observe clinical operations, conduct statistical analyses, and engage directly with CINJ stakeholders to support ongoing research activities, and (c) establishing a sustainable collaboration framework between Rutgers Business School and local healthcare providers, while identifying avenues for future research partnerships. The initiative was supported by an initial grant of $105,000 and included structured monthly meetings to review research progress, along with continuous discussions on scaling the scope and impact of the work. Post-study follow-ups confirmed that all stated objectives were successfully achieved upon project completion.

#### Collaborative structure/mechanisms

The collaboration started by one of the leading physicians, who was then an EMBA student at Rutgers Business School. The physician approached the faculty leading the RBS healthcare program about patient complaints regarding long waits. Press Ganey patient survey data showed delays and wait times were consistently the top two patient concerns in both Q2 2021 and Q1 2022. After analyzing the survey data and CINJ’s processes, the faculty proposed using process analysis and simulation to address prolonged waits and related patient complaints. The team specified key personnels and project deliveries in a memorandum of understanding, which also include the project timeline and the funding mechanism. The key deliverables include the project is (1) to identify bottlenecks for outpatient visits, (2) to develop an optimized workflow based on simulation models, and (3) to create a dashboard as a decision-making tool. The project was led by a faculty from RBS with clinical support from the associate chief nursing officer. Funding was provided to RBS covering assistantships for both the PhD students and multiple master students, who were working for between 10 and 20 h per week from Summer 2022 to Spring 2023.

The timeline for the project was to collect data and conduct data analysis in the first five months (July-November), involving visits to the clinic and observations and documents of patient visits. The following six months were to develop simulation models to mimic patient flows throughout the clinic and to develop a clinic dashboard as a planning tool for nursing officers (December-May). The final month was to collect responses from stakeholders and revise the models and dashboard as needed. It was the expectation that the data from the project will be specific to oncology outpatient visits and hence the models shall consider the clinical nature of oncology services. It was also expected that the project could continue into next year should the need for revision arise.

#### Effectiveness and outcomes

At the beginning of the project, the team spent multiple weeks’ time to visit the clinic and observed its operations during both peak and off-peak times. A flow-map of the clinic, starting with check-ins to vitals, and then to blood draws and therapies, as well as doctor visits, was constructed and revised based on feedback from the associate nursing officer. Before the team conducted the simulation study, multiple teams of graduate students interned with CINJ to observe patient flows, documented waiting times, interviewed both patients and nurses, and conducted initial statistical analyses.

Next, the doctoral student worked with faculty to simulate patient flows with visit data from Electronic Medical Records (EMR). Simulation-based optimization was conducted with OptQuest in AnyLogic, which applies a proprietary “black-box” global optimization algorithm to identify how resources can be optimized to address varying levels of demand, given resource constraints and patient arrival patterns. Three different sets of scenarios were developed based on the requirements from CINJ [36].

The first scenario compares the existing off-site lab setup and the proposed on-site lab setup. Currently, all blood samples from CINJ are sent to an off-site lab for processing and testing, which results in significant delays and extended turnaround time. With the offsite lab, blood results take an average of 58 min with a variance of 34 min. With the onsite lab, CINJ expects turnaround time to follow a triangular distribution (5, 10, 20), where 5 min is the minimum, 10 the most likely, and 20 the maximum. Based on estimated patient demand from CINJ, the team simulated three daily outpatient volumes: the current arrival level, and levels 10% and 20% higher than current. Simulation models developed based on the distribution of turnaround times suggest that visit lengths reduce by 32 ~ 40 min per patient (i.e., average of 15% reduction in visit lengths) and nurse utilization decreases by 10% ~ 13% with the on-site lab.

The second scenario examines how nurse staffing levels affect performance. As total CINJ patient visits are expected to grow, CINJ wants to assess the impact of adding nurses after moving to the new location. Using CINJ’s staffing data, we simulated patient flow for different daily nurse counts and evaluated effects on visit lengths and nurse utilization. Given existing process bottlenecks, the simulation shows that adding nurses substantially lowers nurse utilization across all three outpatient volume scenarios, but the reduction in visit length diminishes with each nurse added beyond the current pool of nine. For example, adding a tenth nurse reduces average visit length by 1–5 min and nurse utilization by about 7%. Adding a eleventh nurse yields at most a 1‑minute further reduction in visit length and a 4–6% reduction in utilization.

The third scenario evaluates alternative scheduling strategies based on CINJ’s recommended operations practice, including a target nurse utilization of 60–70% under projected demand growth. Currently, CINJ books most patients in the morning to address concerns about lab wait times, but this creates bottlenecks given limited therapy chairs and staff. We simulated hourly arrivals and nurse service rates, imposed a 70% maximum nurse utilization and daily capacity constraints, and used OptQuest in AnyLogic to solve the simulation‑optimization problem. The resulting schedule shifts some patients to early afternoon, smoothing arrival peaks and reducing average visit length by 43 min (from 262) while lowering nurse utilization to below 60%.

The optimized simulation results led the clinician team to create fast-tracking schedules to reduce overall waiting times in the outpatient facility. Rather than placing all patients in a single queue, CINJ began creating separate waiting lines for cancer treatment and supportive care, such as transfusions. Through fast tracking cancer treatment patients, CINJ significantly reduced average waiting times and increased the number of daily infusion treatments from 50 to 80 [37]. In addition, a dashboard was also created for clinicians to evaluate performance impacts associated with fast-tracking. The program has produced three conference presentations and a manuscript published with a leading operations research journal [36]. Additional work to apply simulation to improve patent schedules is also under way. Through the funding arrangement, five master’s students gained paid internship experience, through which they engaged with the clinical team to practice data collection and data analysis. The Ph.D. student developed simulation and analytical models for patient scheduling, contributing to his dissertation and presentations at major academic conferences.

#### Lessons learned

We realized several insights from the collaboration with CINJ. First, stakeholder buy-in is critical to successful collaboration with clinical teams. The support from leading physicians and nursing officers is one of the key enablers for the research team to incorporate operations factors into the simulation models in the oncology setting. In general, working with healthcare- and medicine-related collaborators on building computer simulation models can be a frustrating and unproductive exercise for all involved if the collaboration is not established on trust and shared values [38]. Second, settling scope and deliverables early on in the project is essential to ensure a successful completion of the project. Most hospitals and provider groups have multiple competing priorities, including efficiency, patient experience, clinical trials, benchmarks, etc. It is critical for the research team to drive the direction of the project, with consultation from the clinical team.

The initial few meetings for the project were specifically designated for both teams to discuss and brainstorm ideas around what to expect from the project. Articulating project deliverables and hence scope early on helps to lay out a reasonable project plan, which guides further interactions between both teams. A third lesson we learned was that breaking down the project into smaller actionable work packages may be needed to engage different student groups. For Ph.D. students, their project work should lead to potential paper publications and fulfill dissertation requirements. A final lesson is that, given the different working schedules between the research team and clinical team as well as the size of the teams, it is helpful to have a designated project manager to coordinate meetings, keep an eye on project deliverables, and relay messages and other communication in a timely manner.

### Case study 2 – University of Cincinnati Emergency Medicine (Cincinnati)

In 2012, the University of Cincinnati (Ohio, USA) recognized the need and opportunity to create a long-term collaborative relationship between the Department of Operations, Business Analytics & Information Systems (OBAIS) at the Lindner College of Business and the Department of Emergency Medicine (DEM) at the UC College of Medicine. DEM staffs the emergency departments at the University of Cincinnati Medical Center and two large suburban hospitals. This initiative was started shortly after two smaller “one-off” collaborations helped both parties realize what the other brought to the table to benefit the organization, its patients, and the faculty and their students.

#### Primary goals

There were three primary goals for this collaboration: (a) to provide business Ph.D. students with the opportunity to conduct applied operations research in a functioning medical environment, (b) to offer operational insights and data-driven improvement guidance to the department of emergency medicine, and (c) to support the overall research mission of the institution through publication and/or grants. All parties involved have stated that they feel all three of these goals have been met. Programs like this have been openly called for, such as by [24].

#### Collaborative structure/mechanisms

The collaboration was structured as an annually renewable program engaging both departmental units. A memorandum of understanding outlined broad expectations, key deliverables, specific individuals who would be involved from both departments, funding obligations, and annual timelines each year. The key deliverables were stipulated to be research papers and presentations, although other outcomes were produced on a project-by-project basis. The individuals with designated responsibilities were (1) the research director from Emergency Medicine, (2) the faculty research advisor from OBAIS, and (3) the OBAIS PhD student who would serve as the research fellow for that 12-month period. Funding was divided across the two units, with the Lindner College of Business covering the PhD student’s tuition scholarship and the DEM covering the student’s three-semester assistantship, which accounted for roughly 20 h per week over the 12-month duration of each annual agreement.

The timeline stipulated in the agreement created an annual cycle both units could rely on for future planning. The first three months (July–September) of the year-long period were allocated to identifying a suitable research effort. The next three months were intended to be focused on data gathering and statistical/mathematical model-building. The following three months were generally spent on either empirical analysis or model improvement, as well as preparing the research for submission to one or more academic conferences (in both business and medicine). The final three months were spent writing the resulting paper and submitting it to an academic journal, presenting the conclusions to the Department of Emergency Medicine, and potentially discussing opportunities for implementing a trial of the proposed changes within the department’s operating environment. This overall structure, however, was flexible, and several years executed research projects on timelines that purposefully deviated from it (after being reviewed and approved by both departments involved).

#### Effectiveness and outcomes

The program’s effectiveness was evidenced by completed projects, research papers and presentations, and internal operational improvements. Since its inception, the program has produced nearly 30 oral conference presentations, six peer-reviewed journal articles, and three conference poster talks. Research generated via the joint assistantship initiative has also informed the DEM's decision-making in terms of its staffing and shift scheduling policies, designing an acuity-sensitive patient flow, and managing its facility for enhanced operational flexibility. Four different OBAIS PhD students have so far participated in the program, providing them with invaluable insight into the day-to-day workings of a functioning emergency department and allowing them to be immersed in healthcare settings to better appreciate the unique culture of that industry.

One of the first projects that produced both academic and practical benefits examined how flexibility could be strategically applied in the emergency department (ED). The project [39] used discrete-event simulation to model the ED and single- and multi-variable experimental methods to determine what level of flexibility for patient beds was appropriate for varying levels of patient volume, acuity, etc. It found that even a small amount of flexibility (i.e., “partial flexibility”) can offer nearly the same operational benefits of full flexibility. The results of that project helped the department develop rooming and space-allocation policies when designing the new ED facilities that came online in 2023. Building on the experience gained through that project, as well as others that emerged from this collaboration, an additional paper offered insights and guidance to those hoping to employ flexibility in improving ED operations [40]. That paper offered a framework of five different types of flexibility that emergency departments can make use of when modeling and improving their patient flow and efficiency of care delivery.

An aspect of the department’s operations that also benefited from this collaboration is its mobile stroke care team. One project examined how telemedicine can be a useful adjunct to sending teams physically around a region [41]. A Monte Carlo simulation of the stroke team care-delivery system provided guidance as to how the network should invest in telemedicine technology to better balance the benefits to stroke patients against the costs of technology acquisition and upkeep. A broad set of experiences with operational simulation methods via these collaborative projects led to another student-led paper, which offered guidance to those who may be starting to consider using computer simulation to address similar issues [42].

While simulation is common in healthcare operations improvement, we have noticed that optimization methods can be useful if the problem is specified precisely enough. An example of this was a project that sought to develop a model for optimally allocating the emergency department’s physicians’ clinical shifts across three different hospitals so as to achieve a fairer allocation [43]. In this project, we developed a mixed-integer optimization model that balances equality (all physicians get the same deviation from their preferences) with equity (where physicians are prioritized based on the totality of their recent professional contributions to the institution). The implementation of this model led to significant improvements – 31% improvement in physicians' perceptions of fairness in the work-time-allocation process alongside a 51% reduction in scheduling undesirability – demonstrating that unequal outcomes can still be perceived as fair when based on a well-designed decision model.

Other joint projects have resulted in additional papers, presentations, and operational policy guidance. Operations research methods lay at the heart of many other efforts that students, faculty, and physicians have undertaken as part of this collaboration. One side benefit of our work together is that the awareness by the clinicians of the breadth and utility of these quantitative approaches, and of OR/MS thinking in general, is better now than when the initiative first began.

#### Lessons learned

After over a decade of collaboration, several conclusions can be drawn. First, trust and open communication are prerequisites for having a positive and productive relationship. Contributing to that is arriving at a shared vision for Ph.D. students’ development and updating each other as institutional challenges shape and reshape the program. Third, training and culture-sharing can be useful, if not essential. Beyond the business faculty and students’ exposure to the medical culture, we had several training sessions over the years introducing clinicians to operational models and methods, such as optimization, simulation, queueing theory, measurement techniques, and so on. Two surprisingly staunch allies we had within the department were the department chair and the business affairs manager, both of whom had business training and viewed the collaboration quite favorably. Finally, flexibility on both parties’ parts is essential to reducing conflict. In our case, trivial issues such as misalignment between the business school schedule (semesters) and the medical campus schedules (July 1 annual starts) required regular co-management. Also, working together to resolve occasional funding uncertainties and pressures helped greatly extend the longevity of the program. Ultimately, a long-term collaboration requires deep commitment to it from all parties involved.

### Case study 3 – Global Health Innovation Lab at Babson College (GHIL-Babson)

The Global Health Innovation Lab-Babson (GHIL-Babson) is a collaboration between Babson College (Massachusetts, USA), partner universities in low- and middle-income countries, and their affiliate hospitals. The collaboration model (GHIL-Babson Rwanda) began in 2019 between Babson College and the University of Global Health Equity (Butaro, Rwanda), and its affiliated Butaro Hospital and clinics. A second collaboration (GHIL-Babson Brazil) began in 2022 between Babson College and the Unifacisa University (Campina Grande, Brazil) and its affiliated hospital, Hospital de Ensino e Laboratórios de Pesquisa (H.E.L.P.) and clinics (e.g., HVISAO, its ophthalmology clinic).

#### Primary goals

This collaboration has three key goals: (a) to educate undergraduate and graduate students at both institutions in operations and innovation management, with a focus on service delivery and digital health needs, (b) to identify opportunities for improvement and develop testable solutions to pilot with quality improvement and frontline staff, and (c) to develop a culture of improvement and innovation at partner universities and their affiliated medical facilities. These goals have been more fully realized in the GHIL-Babson Brazil model than the GHIL-Babson Rwanda model due to a more stable funding structure that we will discuss later as a mechanism for sustained collaboration. As an international collaboration, GHIL-Babson relates to calls for more operations management research and collaboration in international settings [28, 44].

#### Collaborative structure/mechanisms

The collaboration mechanisms began with annual memorandums of understanding that included key activities and milestones and specification of the lead faculty from both institutions. Funding commitments were also specified to support program development, coordination, and some travel costs; an external foundation supported GHIL-Babson in Rwanda and the philanthropic arm of Unifacisa University supported GHIL-Babson in Brazil. Each institution had a faculty lead and a staff lead. Departmental champions and quality improvement staff represented the clinical interests and proposed projects based on their operational needs. Senior leadership engaged through periodic updates, e.g., Vice President of Quality or the Dean of the Medical School. The key deliverables were: (1) GHIL curriculum development and execution, (2) pilot implementation of the identified improvements within each participating department, and (3) a final presentation video and white paper summarizing the process and outcomes of each project.

GHIL-Babson includes a Fall operations and innovation management course that includes one week of in-person observations for participating Babson students in the partner country followed by weekly virtual class sessions; a Spring implementation-focused application phase; and a Summer Fellows program to continue implementation and source the next round of projects via interviews and data collection. Throughout the year, Babson students (undergraduate business administration, Master of Business Analytics, and MBA students) and partner university students (primarily undergraduate medical and IT students) form teams to develop and test solutions to five operational challenges identified by hospital and clinic champions.

The GHIL-Babson course teaches students Value Sensitive Design and advanced Quality Improvement techniques, and they apply these techniques to their projects. Value Sensitive Design (VSD) is “a theoretically grounded approach to the design of technology that accounts for human values in a principled and comprehensive manner throughout the design process” ([45], p. 69). Quality Improvement (QI), in the context of health care, is an iterative, continuous, data-driven process to improve the delivery of care and reliably satisfy or surpass patient needs and expectations [46]. As a capstone styled course, students are expected to apply the most appropriate techniques from their introductory and intermediate operations management courses for their challenge (e.g., economic order quantity examples in pharmacies, ambulance routing models, or patient notification systems to reduce no-shows and late cancellations). Students conduct two iterations of design and improvement, where each iteration includes developing or refining a problem definition and point of view statement, clinician interviews and data collection, ideation and solution development, and testing.

#### Effectiveness and outcomes

Because GHIL-Babson predominantly engages undergraduate students, our primary aim is to provide an effective educational experience. Notable outcomes included high student participant satisfaction (96%) and increased student innovation self-efficacy (97% reported increased innovation self-efficacy). The course has been recognized via multiple teaching awards including Babson’s Dean Award and the POMS Wickham Skinner Teaching Award.

Each year we aim to have at least one of our five projects fully implemented within clinic operations. For example, one GHIL-Babson Brazil project focused on reducing no-shows, late cancellations, and cancellations due to patients who have not done the appropriate pre-surgical preparation steps for their retinal surgery appointments. Because the partner organization had limited historical data, students used interviews and direct observations to estimate that four surgeries were not conducted per month in one eye clinic due to no shows and late cancellations, resulting in ~ *R$224,000* (or ~ $42,560 USD) of lost revenue annually. As explained by one of the nurses, “Even one less cancellation would have a big impact on a patient's quality of life.” There was also opportunity to implement the solution at a second eye clinic within the health system with similar challenges. The students found that elderly and illiterate patients were more likely to no-show or cancel. The students designed analog and digital solutions, including a comic-strip styled brochure and a WhatsApp texting system of audio, video, and written reminders. They conducted testing with 15 patients and 7 providers. The WhatsApp notifications from pilot project continued within the clinic. Clinical staff reported one fewer no-show per month on average and that these notifications increased patient awareness of their pre-surgical preparation steps.

A project through GHIL-Babson Rwanda developed an ambulance-routing system for the Butaro District of Rwanda. The student team used available data to determine the current ambulance dispatch time was 47 min from call to arrival to patient compared to 7 min on average in the U.S. They developed a route-optimization algorithm that reallocated the positioning of the ambulances. The resulting model reduced dispatch times by half. This solution was first shared with the local Butaro Hospital project champions who shared the findings with district and national leadership. By the end of the project, the broader national ambulance dispatch committee was developing a nation-wide solution. While our students’ specific solution was not fully implemented, we believe this project generated awareness that informed action.

#### Lessons learned

First, as the primary objective of GHIL-Babson was to provide operations and innovation management education using an experiential approach with industry partners, we learned that there were equally valuable lessons regarding cross-cultural teamwork that required supplemental tools and activities. Some students with English as their second language reported challenges with actively participating in discussions during lectures; closed captioning and transcriptions of virtual classes and meetings were encouraged to support language comprehension. Some students reported difficulties collaborating with their teammates. Over time, we required teams to tour important cultural sites, museums, and participate in arts activities together in-country. Then, faculty led discussions to review the experiences, noting cultural differences such as the relational-orientation of Brazilian partners versus the task-orientation of US partners. Informal meetings with faculty allowed students to express differences in working styles. Faculty also joined informal student team meetings and group chats to provide more guidance as needed. These additional tools and techniques were reported to support stronger team relationships, communication, and bridge cultural differences by students and clinical project champions. While these cross-cultural differences were observed across participants from different countries, similar techniques could be considered to address the aforementioned conflicting cultural differences between operations research students and medical professionals (e.g., closed captioning and transcriptions to record medical language in meetings; touring hospital displays to understand historical context).

Second, as we seek to identify improvement opportunities and testable solutions, we learned to reduce the scope and more clearly bound the problems proposed by clinicians to improve the likelihood of a feasible project pilot. For example, reducing antibiotic misuse was an early project in GHIL-Babson Brazil that leveraged data analytics to identify trends and standard use design for improved physician communication (as per [47]). Because transformation required the input of multiple stakeholders beyond the scope of the collaboration, it was difficult to assess the effectiveness of the proposed solutions. Therefore, in subsequent years, projects have focused on incremental improvements selected by the quality improvement teams to prioritize feasibility and real-world relevance.

Finally, funding can be an ongoing challenge, particularly as it relates to the longer-term goal for GHIL-Babson to support a culture of improvement and innovation at partner universities and their affiliated medical facilities. GHIL-Babson Rwanda unfortunately ended after four years as it was supported by an external grant that was not renewed. GHIL-Babson Brazil continues in part because of internal funding support from the partner institution’s affiliated foundation and consistent support of the hospital leadership. Having a combination of institutional and external support can help to address this challenge.

### Case study 4 – Michael E. DeBakey VA Medical Center in Houston, Texas (MEDVAMC)

In 2009, the Michael E. DeBakey Veterans Affairs Medical Center (“the hospital”) in Houston (Texas, USA) sought academic collaborators to help address operational issues in its perioperative department, including significant challenges related to scheduling and process inefficiencies, resource allocation, and patient flow management. This collaboration involved faculty from the University of Houston (UH) and the Baylor College of Medicine (”Baylor”), as well as undergraduate and graduate students majoring in logistics and supply chain management.

The collaboration started as a casual interaction between the faculty in the supply chain and logistics department at UH with the Baylor-employed hospital surgeons. The primary investigator (PI) from UH was trained in operations research methods and the proposal they made was accepted and then funded by the hospital. The funding mechanism was direct annual payments from the hospital to UH. This allowed UH to let the PI focus on research (with reduced teaching load) and employ students, as needed, throughout the various phases of the project (discovery, data collection, data cleaning, analysis, report outs).

#### Primary goals

The primary goals of this collaboration were to (1) rigorous evaluation of perioperative processes to identify sources of inefficiencies; (2) provide UH undergraduate and graduate students with practical experience in health care operations, and use that as their final practicum; (3) leverage academic expertise to improve operational efficiency, and ultimately enhance patient outcomes; and (4) provide opportunities for mid-career faculty at Baylor to learn operational management skills, as well as to participate in funded research. The collaboration aimed to address these challenges by applying advanced operations concepts and analytical models and methods.

#### Collaboration structure/mechanisms

The collaboration was structured around a series of targeted projects, each focusing on a specific aspect of perioperative operations. Meetings were held weekly in the initial phase, and then once a month to discuss project progress, share insights, and alignment on goals. The students and faculty (from the Baylor College of Medicine who are deployed to the VA Medical Center) worked closely with perioperative staff, including surgeons, anesthesiologists, nurses, and administrative personnel, to understand the existing processes and identify areas for improvement.

A unique aspect of the collaboration was the insistence from the hospital to have the PI embedded in their organization. This was because the hospital’s previous engagements with consulting organizations had not been satisfactory on account of the consultants’ transactional relationship with the organization. The hospital was therefore interested in a collaborative research-oriented engagement. The PI was provided with access to the hospital’s staff, data, physicians, and an office space. The PI was invited to meetings where perioperative efficiency discussions were held. This helped the research team to develop solutions keeping the real-world constraints front and center.

#### Effectiveness and outcomes

The collaboration resulted in several tangible changes and improvements in the perioperative department. Key outcomes included the development of a predictive scheduling model that reduced patient wait times and optimized the use of operating rooms. Additionally, resource allocation was streamlined, leading to a more efficient use of staff and equipment. The collaboration also provided valuable hands-on experience for the students involved, enhancing their understanding of healthcare operations and preparing them for future careers in the field.

The hospital caters to a unique population (veterans) that often have no other avenue to seek care. They are also often geographically dispersed and far from the main hospital, which makes appointment scheduling challenging. Most such veterans live far from the main VA hospital and have to be incentivized to come a day prior to their surgery and be housed in hotels to ensure a timely start of the OR-day next day. The initial analysis of the clinic appointment schedules and surgery cancellation rates revealed that most of the solutions published in the extant research in operations research and management may not apply to this population. Creating metrics through retrospective/descriptive analytical tools to scope out the issues itself was very insightful and useful for the hospital leadership, as it validated quantitatively what they were experiencing first-hand. The analysis revealed that there were issues like case cancellation rates (15% +, compared to 3–5% in academic medical centers), that were beyond control, however, what could be made more efficient were operating room turnover times, case length prediction and case scheduling. The assessment also helped delineate issues related to capacity planning (longer term plan) vs. capacity management (day-to-day processes that influence capacity utilization). In became clear that capacity planning decisions (such as, how much capacity/blocks to assign to which surgical specialty) were beyond the scope of this engagement as those decisions are based on many different strategic priorities (even though from a purely operations perspective they may be considered sub-optimal). It also became clear from timestamp mapping that the day-of-surgery processes could be improved through better real-time information sharing and improved coordination and incentives. The hospital decided to pilot a Real Time Location System to help improve on-time starts and turnover times.

The greatest opportunity to deploy rigorous operations research and management tools was in the mid-term capacity management decisions (decisions that are made within 2 weeks of surgery). Initial assessment of patient flows through the perioperative arena identified two key areas of focus: improving predictability of surgery duration and improving scheduling efficiency. In the initial discovery phase of the project, using standard statistical techniques, the team figured out that entire duration of a patient’s time in the operating room can be decomposed into “wheels in” to incision” (about 20 min for simpler surgical cases such as ophthalmology and general surgery cases like hernia), “incision to close” (also called surgery time, which varies depending upon procedure: 30 min for most Ophthalmology cases, around 4 h for cardiac surgery cases, and up to 10 h for craniotomy (brain tumor) cases), and “close to wheels out”. It was also determined that the greatest variability is in the surgery time, which is surgeon-procedure dependent. Although this had been studied before, the analytical and rigorous analysis was appreciated and resulted in several publications in medical journals [48, 49]. It was found that irrespective of surgeon experience, some surgeons take longer to do the same procedure, without negative patient outcomes. The surgery duration most appropriate for scheduling a case is therefore the surgeon’s previous experience on that procedure. Most Electronic Medical Records systems (such as Epic Systems) now use the surgeon’s time on the same past five procedures to prompt the surgical scheduler when scheduling that procedure.

After establishing better ways to predict surgery duration, a theoretical exercise using simulation modeling was undertaken to devise a more efficient scheduling technique [50]. The simulation showed that either more vascular surgeries can be scheduled on average in an operating room (15%), or the end of the day can be more reliably determined using the surgery-duration prediction model (23% using the simulation model vs 34% using averages). These studies then became the foundation for the bigger scheduling efficiency project. In this, the prediction models built earlier were tested through a funded randomized control trial and their efficacy to improve daily schedules of operating rooms was demonstrated [51]. The trial ran 207 days, alternate days were scheduled either the traditional way (averages) or the modeled way (regression-based on predicted time for surgeon-procedure combination). Mean imprecision in predicting the end of the operative day was higher with the averages-based approach (30.8 vs 7.2 min, *P* = 0.024). Regression-based days were associated with higher throughput (379 vs 356 cases scheduled over the course of the study, *P* = 0.04). The composite rate of adverse 30-day events was similar (2.2% vs 3.2%, *P* = 0.44). In addition to quantitative metrics, qualitative scoring of staff’s satisfaction was also done using the modified Maslach Burnout Inventory survey. The mean depersonalization score was higher (3.2 vs 2.0, *P* = 0.044) and mean personal accomplishment score was lower for weeks that used averages-based scheduling (37.5 vs 40.5, *P* = 0.028).

#### Lessons learned

Several critical lessons were learned from this collaboration. First, the importance of stakeholder engagement was evident, as the success of the projects relied heavily on the active participation and support of perioperative staff. Second, the need for clear communication and regular updates was underscored, ensuring that all parties remained aligned and informed. Finally, the collaboration highlighted the value of applying academic research to real-world problems, demonstrating the potential for significant improvements in health care operations through such partnerships.

The timescale over which academic research typically develops can be less compact than the time-critical nature of most real-world problems in this industry. For example, when health care delivery organizations seek collaboration with academic institutions, they often have issues for which they want solutions in a relatively immediate time-period (weeks or a few months). However, merely identifying and developing interesting and unique research questions can take that amount of time, let alone answer them. So, how should this conundrum be resolved? Two lessons were learned. First, active involvement of the practitioners in the process helps them understand and appreciate the analytical way in which operations research and operations management approach and dissect the issue. During this process, the academic researcher should remain open to practitioner feedback and keep focus on the real-world problem. Practitioners benefit from this continuous involvement as they better understand the nuances of modeling and its inherent assumptions. Second, undergraduate and master’s students can play significant roles in helping solve the practical issue within the shortened time expectation of the practitioners (if effectively managed). The academic faculty and Ph.D. students can then tackle the more scholarly (i.e., more generalized and/or more theoretical) version of the problem over a longer time scale. This approach produced a win–win in this particular engagement.

### Other models and examples

While these case studies illustrate a variety of approaches, they are by no means exhaustive in cataloging the ways operations academia and the healthcare industry can collaborate. This section offers a variety of additional models that may fit the needs of a particular situation better than the approaches described above. We rely on the framework in Fig. 1 above to help organize these approaches.

In Fig. 1 (above), type “a” collaboratives involve healthcare institutions and recent doctoral graduates (only). These efforts often produce arrangements similar to traditional post-doc appointments, but without the teaching responsibilities, and can be quite valuable to both parties involved. Not only does the institution get access to a junior scholar with valuable analytics skills and state-of-the-art knowledge of their field, the post-doc will usually get access to data that are usually off-limits to anyone outside the institution as well as access to meetings and discussions that can motivate unique and timely research questions. Having clear expectations, timelines, and funding mechanisms outlined in advance will be helpful to maximize the performance of such arrangements.

In Fig. 1, type “b” collaborations involve the care delivery organization, one or more faculty members, and one or more doctoral students. Case 2 (Cincinnati) above provides a detailed example of this sort of collaborative model. Efforts falling into this category can often start as one-off efforts, where the faculty bring(s) on the PhD student(s) to assist in various steps, such as process analysis, data gathering, and analysis/modeling. Making these arrangements into long-running arrangements (i.e., extending beyond a single project) can require sustained investment by the healthcare delivery organization as well as advanced planning by the faculty involved, since doctoral students may not be available consistently for more than a year or two. As a result, developing a pipeline of students interested in this area and capable of contributing to it is essential.

Type “c” in Fig. 1 includes collaborations between a healthcare institution/provider and one or more academic faculty (only). Two examples of this type of arrangement include sabbaticals (where a faculty member spends an amount of time, such as a semester or a year) embedded within a healthcare facility or institution to learn in situ), and joint and courtesy faculty appointments (where faculty are granted enhanced access to meetings, personnel, data, and facilities through an additional formal institutional position). Both can be valuable positions from which long-term, collaborations with more comprehensive scope may be engineered. For example, a portion of an ongoing collaboration between Stanford Engineering and Lucile Packard Children’s Hospital Stanford included faculty with joint appointments between the institutions at the time of publication, leading to three machine learning projects focused on generalizable, EMR-based clinical decision support tools in cardiac care: false alarm suppression, detection of critical events, and automated identification and detection of drug-drug interactions [15].

While rare, some business and engineering faculty have taken on full-time primary appointments in health care-related departments and institutions. While this approach raises tremendous challenges (e.g., how publications and grants are valued by the department versus a department in the faculty member’s home field), it can facilitate learning for the faculty and translation from theory into practice on a scale that many traditional appointments simply cannot achieve. Another rare type “c” collaborative arrangement that can occur is a government agency appointment, where a faculty member may temporarily take leave from their academic position to work in/for a governmental or quasi-governmental organization involved in health care delivery and/or policy-setting.

The most comprehensive of type “c” collaborations may result in distinct organizational entities, such as institutes or divisions, dedicated to focusing academic research faculty efforts on improving the day-to-day operations of a partnering health care delivery facility or network. Such an initiative, for example, resulted in the Institute for Health Care Delivery Science at the Icahn School of Medicine at Mount Sinai [26]. The documentation of that institute’s origins and evolution describes how funding and overcoming cross-disciplinary hurdles continue to be ongoing challenges, common for such quasi-self-standing organizational units. Another variant on this approach may have an industrial entity other than a health care provider acting as the prime mover driving the collaboration. Such a situation, where Philips Healthcare worked with academic and health care partners to establish a health data clearinghouse and research environment, was documented by [52]. In another, MARS, a large multinational company headquartered in the United States, co-located technical leaders on the UC Davis campus, developing several research initiatives including the Innovation Institute for Food and Health that develops future innovator talent in food and health and uses new technologies to innovate healthier foods [28]. Generally, academic-industry collaborations focused on health informatics are increasingly common as the volume and complexity of health-related data continues to increase [53].

A common first attempt at collaboration between academic faculty and health care institutions is the one-off research project (e.g., [31]). Often these efforts come about by an expressed need within the institution intersecting with the expertise and availability of a faculty member. These arrangements can be of types b, c, d, and e in Fig. 1, depending on whether they involve doctoral students, master’s or undergraduate students, both, or neither. While these one-off, limited-scope efforts can lead to publications for the academics involved and can provide useful information for the care delivery organization, if the relationship ends there, much opportunity and upside potential has been squandered. But when collaborators build upon the initial project, it can be quite productive for all parties. For example, the collaboration between Massachusetts General Hospital (MGH) and the Massachusetts Institute of Technology Sloan School of Management began with a project in 2011 to optimize surgical flow by smoothing out the weekly patient census on the surgical floors. This collaboration has now spanned 15 years, leading to the engagement of several faculty and students, numerous articles published and data-driven implementations completed, and is managed by the MGH Vice President for Healthcare Systems Engineering (a role constructed in part because of the collaboration), with direct guidance, funding, and oversight from hospital executives [14].

Another category of collaborative arrangements that is similar includes those involving faculty consulting, with or without publishing possibilities, also potentially occurring in areas b, c, d, and e in Fig. 1. While the model discussed in the previous paragraph expressly involved research, this approach tends to start off as an operational improvement project with no clear path to a research publication. These efforts – often referred to as quality improvement (QI) initiatives – tend to be motivated by a very real and persistent aggravation within the healthcare provider. In a hospital, as an example, that could be left-without-being-seen (LWBS) rates (i.e., the proportion of patients who check in but leave before receiving care) that are higher than the institution desires. The hospital could approach an operations faculty member to help it develop creative and theoretically justified approaches to fighting this problem. Only after the project is complete might the faculty member realize that there are generalizable research findings potentially suitable for publication. Because these efforts start with no direct relevance to research, they can be deemed less important by faculty who are still seeking academic promotion. But these projects, if successful, can often lead to more symbiotic relationships that help the faculty member pursue formal research efforts that previously would have been difficult to initiate.

Finally, co-ops, internships, and other work-study arrangements for undergraduate and master’s students – area “f” in Fig. 1 – represent an opportunity for many students who might be interested in applying operations knowledge to healthcare problems. The Veterans Affairs Engineering Resource Center is one example, where their Fellowship Programs provided training to engineering and clinical staff on advanced industrial engineering topics and methods from affiliated university-based engineering faculty; Fellows also conducted self-directed learning from assigned individual and team projects [16]. As these expand the scope of academia-industry collaborations, they are valuable to both the healthcare institution and to the students who receive such opportunities. Indeed, such students can often become invaluable members of research teams should they go on to pursue advanced training in operations-related fields.

## Discussion

These case studies and other models illustrate many of the ways academic collaborations with the healthcare industry can be structured and the significant benefits they can provide. In this section, we attempt to synthesize these case studies with the limited literature available and offer useful guidance to those who may be considering initiating such a collaboration or wondering how best to sustain or grow an existing one.

### Collaboration maturity

Collaborations often evolve along a maturity life cycle, with more successful and long-lasting collaborations evolving further than those that are more limited in duration or scope. The first level of maturity is the transactional collaboration. These tend to be limited in scope, often focused on investigating or improving a specific problem (which can often begin as a request for a solution rather than help exploring the root cause of a problem). They tend to have minimal formal structure and often resemble one-off consulting projects or student appointments. Many research projects in the operations literature began as just these sorts of collaborative efforts.

The second level of maturity is the formally structure project. This tends to be a fixed-term, formalized relationship (e.g., Rutgers-CINJ above), often involving a clear memorandum of understanding (MOU) with defined deliverables, timeframe, funding arrangements, and so forth. They are more focused on metrics and measures associated with project completion than with building the collaborative relationship.

The third level of maturity is the renewable partnership. These collaborations often involve annual review of ongoing commitments towards a longer-term set of objectives and goals (e.g., Cincinnati, GHIL-Babson, and the VERC initiative mentioned above). They have an established rhythm of deliverables and expectations, often serving as an umbrella arrangement covering multiple parallel and/or serial projects. The individuals involved in these efforts may be larger in number and more diverse in their roles than at lower levels of collaboration maturity. These require trust and shared understanding, as they may need to survive leadership changes, and require predictable funding cycles supported by both sides of the arrangement. From our experiences, building such trust cannot be prescribed; rather, it is created by many factors, especially by having a sensitivity to the communication style and organizational culture of each particular collaboration partner. Activities and efforts that foster trust can be invaluable in the long run.

The fourth level of maturity we observe is the embedded/institutionalized collaboration. This level of maturity is exemplified by deep integration, with operations faculty embedded within the health care delivery organization in some long-term form (e.g., MEDVAMC). There may be dedicated office space, standing meeting invitations, and organizational responsibilities allocated across the collaboration line. This goes beyond a long-term commitment, potentially shaping how both organizations view critical, strategic decisions such as hiring, funding, and even physical infrastructure investments. These create the opportunity for permanent third entities, such as institutes or centers (e.g., Mount Sinai).

Collaborating entities that wish to evolve their arrangements along this evolutionary continuum must invest in the relationships and accept the fact that, like every speculative undertaking, not every initiative will necessarily bear useful fruit. Turning short-term teams into long-term relationships requires deep commitment, clear communication, a shared vision, and trust.

### The value proposition triangle

The value produced by these collaborations seems to occur in three primary categories: Operations, Research, and Education. Operations value and benefits are those associated with better and more efficient/timely care delivery, such as through improved patient flow, staff scheduling, resource utilization, and other common metrics. Research value and benefits are first and foremost generated through publications, but can also take the form of patents, commercialized inventions, conference presentations, and white papers. Education value and benefits fall largely in the domain of student development, such as through the experiential value of seeing theory put into practice (or not) and the cross-disciplinary exposure to the health care field’s unique culture, language, and values.

Operations-dominant collaborations, such as the MEDVAMC case above, tend to balance practitioner urgency with research rigor, sometimes using students to work on “quick wins” for more immediate operational improvement. A challenge inherent in operations-dominant collaborations is that the urgency-driven quick win, while valuable to the care organization, may leave the academic partner without the systematic baseline data or controlled conditions needed for a peer-reviewed publication. We encourage scholars to try to anticipate this tension before a project begins or, at minimum, capture whatever retrospective baseline data the organization's existing records permit. When prospective baselines are unavailable, surrogate benchmarks from the published literature can sometimes be used to contextualize results, as can descriptive statistics drawn from the pre-intervention period in administrative records. For those quick-win projects that reveal unexpectedly significant findings, several outlets exist for practice-oriented scholarship, including *Health Care Management Science*, the *INFORMS Journal on Applied Analytics* (formerly *Interfaces*), and *Health Systems*.

Research-dominant collaborations, on the other hand, focus primarily on academic work products, with improvements to practice having secondary value when possible. An example of this approach is the Cincinnati case above, which permitted longer publishing lead times and a more flexible definition of project “success.” Education-dominant collaborations, as illustrated by the GHIL-Babson case above, focus primarily on the needs of students and often attempt to maximize the number of students able to benefit directly from the arrangement. Teaching awards and educational products tend to be key deliverable metrics. There can also be hybrids of these three value proposition archetypes, with any combination being potentially feasible. For example, the Rutgers-CINJ case above balanced the care delivery organization’s needs for a fast-track development and implementation of an improvement effort with both the need to make a useful student experience and various research publications over a multi-year period. Ensuring that the collaboration knows what type of value proposition it needs to produce in advance can be invaluable to ensuring aligned expectations as the relationship unfolds and, ideally, grows.

### Success factors and benefits

Key factors contributing to successful collaborations include stakeholder engagement, clear communication, and the application of academic expertise to real-world problems. Each case study also highlights the educational value of such collaborations, providing students with practical experience and preparing them for future careers in health care operations. The case studies reveal that trust and stakeholder buy-in are foundational. All four cases emphasized this, with MEDVAMC contrasting the embedded relationship with previous transactional consulting engagements that were not as fulfilling or productive. Building trust requires establishing clear scope and deliverables early. Rutgers-CINJ used explicit deliverables in their memorandum of understanding, while GHIL-Babson learned that focused initiatives outperformed overly broad projects. Structured communication, such as through monthly meetings, designated project managers, and regular reporting, ensures ongoing alignment.

Flexibility and realistic time horizons often separate sustainable partnerships from short-term, one-off projects. MEDVAMC strategically deployed undergraduate and master's students for quick operational wins while allowing PhD students and faculty to pursue deeper research, addressing the tension between academic timescales (years) and practitioner urgency (weeks). Moreover, if the collaboration produces operational improvements that are implemented, making those improvements permanent will likely require long-term organizational commitment and/or resources, such as training, manpower, and funding. Ideal collaborators commit to the discovery journey, embrace rigorous research processes, maintain aligned incentives, understand methodological limitations, and communicate clearly. Successful collaborations often reveal connections beyond the immediate organization, such as to government agencies, NGOs, or commercialization entities. GHIL-Babson's ambulance routing project, though not fully implemented locally, informed national-level solutions. However, leveraging these dimensions requires time that may not yield traditional publications.

Across the cases, we note that personnel configurations can vary strategically. PhD-intensive models (Rutgers-CINJ, Cincinnati, and MEDVAMC) prioritize research outputs, while master's/undergraduate-intensive approaches (GHIL-Babson) emphasize implementation capacity. Similarly, funding mechanisms can vary widely, including split-institutional funding, direct grants, foundation support, and embedded models, though sustainability can remain a persistent challenge.

Finally, successful collaborations tend to have incentives that are aligned with both the operations scholars (in terms of publication, timing, and implementation goals) and the care delivery organization (e.g., quality, access, and efficiency improvements). Ideally, the clinical partners are willing to learn enough about the requirements and limitations of the research methods being used to avoid unrealistic expectations. While business and engineering academics sometimes lament that their industry partners are not sufficiently keen on learning about the science of operations, health care practitioners are often surprisingly interested in becoming more educated on these topics. The Cincinnati case explicitly incorporated training sessions to introduce clinicians to operational models and methods, finding that awareness of the breadth and utility of these quantitative approaches improved significantly over time. Health care professionals have often become used to rapid uptake of new ideas and information, as the science of medicine is in a state of constant change. As a result, collaborating with health care professionals can often be surprisingly gratifying, as there is often a faster recognition of the potential value that OR/OM/MS faculty and students can offer the medical facility and its staff and patients.

Collaboration sometimes takes the form of a one-on-one, one-and-done research project, but successful collaborations often reveal layers both within the health care organization and beyond it. For example, the practitioner with whom an operations scholar works may have connections with a government agency that sets policy far outside the hospital walls, or is connected to an NGO that can implement on a broader scale or in another region, or with a commercialization entity to help disseminate the scholar's research via industry products and services. GHIL-Babson's ambulance-routing project in Rwanda illustrates this phenomenon: while the student solution may not have been fully implemented at the local hospital, it generated awareness at the national ambulance dispatch committee level. Understanding and making use of these additional dimensions can require an investment of time and may not pay a return measured in traditional high-quality journal publications. Whether that is a valuable use of the faculty member's time depends on the institution and their particular career objectives. A shortfall in trust, communication, scope clarity, flexibility, or mutual learning can create unnecessary friction, potentially undermining the viability of the entire collaboration.

### Risks and challenges

Despite the benefits, academic-industry collaborations face several challenges. Key challenges include differences in organizational culture, misaligned goals and expectations, and logistical issues such as funding and scheduling. The cases reveal that funding sustainability remains an ongoing challenge; Cincinnati required continuous negotiation, while GHIL-Babson's international collaboration faced travel costs that contributed to their Rwanda model ending after four years. Funding mechanisms varied considerably, including bi-institutional arrangements (Cincinnati), significant direct grants (Rutgers-CINJ), foundation support (GHIL-Babson), and embedded models with reduced teaching loads (MEDVAMC), but all required sustained commitment and creative structuring.

Scope management proves particularly difficult given competing hospital priorities, such as efficiency, patient experience, clinical trials, and benchmarks, requiring academics to "drive the direction with consultation" from clinical teams while remaining responsive to organizational needs. Expectation alignment requires ongoing effort, as GHIL-Babson discovered when better briefing of project champions significantly improved outcomes by clarifying what students could realistically deliver within the given timeframe. The tension between publication and implementation presents another fundamental challenge. Academics need generalizable findings suitable for peer-reviewed journals, while practitioners need solutions that work within their specific constraints and that (ideally) can be deployed quickly. This makes it difficult to generalize findings beyond the immediate context, often limiting theoretical contributions.

Moreover, these collaborations typically require significant time and energy investments. For junior scholars, dedicating 6–12 months to meetings before any substantial work is completed can be impossible to justify when facing tenure pressures. Becoming fully informed and adept at collaborating with healthcare organizations often requires five to seven years in regular communication. This lengthy inculturation period can be particularly challenging for untenured faculty if the collaboration is their sole source of research opportunities. A strategic approach to mitigate this risk is to include such collaborations as part of a broader portfolio of research efforts, thereby diversifying activities and reducing the impact if individual collaborations do not lead to publications. Pairing up junior faculty with more senior faculty who are already experienced in this type of collaboration can be both effective and developmental.

Before embarking on healthcare industry collaboration, academics must carefully consider how such efforts will be valued by their departments. While some institutions encourage these collaborations, others prioritize traditional scholarly outputs over collaborative work. Pre-tenure faculty should assess whether engaging in such collaborations might adversely affect their research productivity and standing. A frank discussion with one's department leadership when starting to consider this path can help navigate these issues. Additionally, misaligned incentives between academic researchers and health care practitioners can undermine collaborations. For instance, if a health care organization prioritizes grant funding while a business school does not value grants similarly, this misalignment can lead to uneven motivation and contribution, potentially causing resentment and breakdown. Successful collaborations require that benefits are shared across the team rather than concentrated in individual researchers or entities.

### Additional recommendations

Beyond these observations, we also offer some additional guidance based on our experience. Academicians aspiring to collaborate with the health care industry should learn the industry's language and culture and reflect unique aspects back to them. The influence and importance of *context* in health care settings is unavoidable [54, 55]. Don't force academic norms and jargon onto the relationship initially. Start with practical problems the industry already suffers from rather than hypothetical problems that align neatly with an academic's methodology of choice.

Also, respect practitioners' experience-based knowledge and listen carefully; practitioners have often already accumulated decades of experience managing and improving the systems that continue to cause them frustration. In some cases, the most pervasive problems have become invisible to those dealing with them daily, making them ripe opportunities for academics who can point them out compellingly. Look for win-wins that solve industrial problems while generating value for researchers and students, though quick wins for organizations can generate goodwill leading to longer, deeper collaborations.

As hinted at above, it can be wise to start with practical/real problems the industry already suffers from rather than hypothetical problems that may just happen to line up neatly with an academic’s methodology of choice (or, as one of the authors remembers a senior OM scholar saying in a recent meeting, “the hospital is not a basket of nails just waiting for your hammer to come along”). While focusing first on the specifics of a real problem (versus the relevant theoretical concepts) may seem frustrating to an academic, it is useful to realize this is vital to developing one’s inductive reasoning skills, which are essential to working with industry partners.

Finally, invest in your colleagues’ and students’ ability to interact with and manage these collaborative relationships. Very few, if any, doctoral programs systematically expose their students to cross-disciplinary situations, with even fewer giving them managerial responsibility. Training, if even rudimentary, in the skills needed to build and maintain these relationships and systems could lay the groundwork for many successful collaborations in the future.

### A Call to academics

Ultimately, academic collaborations with the health care industry offer significant potential for improving operations, advancing research, and providing valuable educational experiences. By drawing on the strengths of both sectors, these partnerships can drive innovation and lead to meaningful improvements in patient outcomes and operational efficiency. The case studies provide valuable insights into factors contributing to successful collaborations and challenges that must be navigated. By fostering a culture of collaboration and leveraging the unique expertise of each sector, academic and industry partners can work together to address the complex challenges facing health care.

In the introduction to this paper, we mentioned “a general call for operations researchers to invest in these collaborative efforts in order to enhance both the usefulness and theoretical relevance of our scholarly endeavors.” We stand by this request to our academic colleagues to reach across the academia-industry barrier to foster such relationships. The value to practice, to theory, and to our students cannot be denied. While the benefits may likely be enjoyed only in the long-run, today may indeed be the best time to lay the groundwork for creating both value and knowledge in the future.
